# Parenthood—Lost and Found: Exploring Parents’ Experiences of Receiving a Program in Emotion Focused Skills Training

**DOI:** 10.3389/fpsyg.2021.559188

**Published:** 2021-06-03

**Authors:** Nadia Ansar, Aslak Hjeltnes, Signe Hjelen Stige, Per-Einar Binder, Jan Reidar Stiegler

**Affiliations:** ^1^Norwegian Institute of Emotion-Focused Therapy, Bergen, Norway; ^2^Department of Research and Development, University of Oslo, Oslo, Norway; ^3^Department of Clinical Psychology, Faculty of Psychology, University of Bergen, Bergen, Norway

**Keywords:** emotion focused skills training for parents, emotion processing, experiential therapy, thematic analysis, emotion focused therapy

## Abstract

**Background:**

Parents play a crucial role in the development, maintenance, and deterioration of child difficulties. Emotion focused skills training (EFST) targets parents’ capacity to provide their child with emotion-oriented skills in order to promote good child mental health. Few qualitative studies have specifically investigated parents’ experiences of receiving such programs.

**Objective:**

This study aimed to explore how parents experience working with their own and their child’s emotions undergoing a short-term program in EFST; in particular, changes in their experience of being a parent and in everyday life are reported.

**Method:**

Semi-structured in-depth qualitative interviews were conducted with 14 parents who had completed a short-term EFST program (2-day group training and 6 h of supervision). Interview transcripts were analyzed using a reflexive thematic analysis approach.

**Results:**

A total of 14 parents (40% men, four couples, *M*age = 39.5, *SD* = 4.4) participated in the study. Our analysis resulted in the following three themes: (1) “Coming home” as a parent, with the following subthemes: (a) New ways of being with their child and (b) Parents’ painful inner world; (2) Reclaiming parenthood—applying new tools and learning in challenging situations; and (3) This is us—changing the heart of the story. The first theme was related to the descriptions of the changes that emerged in parents’ inner lives, the second revolved around the employment of their skills intuitively and creatively based on what was required by the challenging situations, and the third theme referred to new discoveries on family dynamics.

**Conclusion:**

Parents’ experiences of having wisdom and calmness inside them (*being*) and *doing* parenting differently, as well as the changed perspectives of the family (*living*), resonate with the theoretical ground of emotion-focused therapy (EFT). The findings also indicate that therapists should be aware of potential parental distress when working in view of changing unpleasant emotions in such skill-based programs.

## Introduction

Children’s mental health symptoms have been associated with poor emotional competence and difficulties in emotion regulation (Duncombe et al., 2016). Parents play a crucial role in the development, maintenance, and deterioration of these difficulties (Gottman, 1998; Ginott, 2003; Yap et al., 2014). Recent studies demonstrate the significance of parents’ active involvement in the child’s recovery process and indicate an empirical relationship between parenting styles and mental health difficulties in children (Reitz et al., 2006; Mackler et al., 2015; Roskam et al., 2016). Interventions that focus on parents’ capacity to provide their child with emotion-sensitive skills promote good child mental health and prevent the development of emotional and behavioral problems (Havighurst et al., 2013). Parental qualities, such as self-efficacy, warmth, emotional awareness, and acceptance, act as protective factors (Greenberg, 2015; Kårstad et al., 2015; Ford et al., 2017). Parental emotional regulation and their ability to accurately respond to their children’s emotional states can be even more decisive in predicting child emotional development (Vikan and Kårstad, 2012; Reinfjell et al., 2015). Parental belief about children’s emotions and their ability to correctly perceive their child’s comprehension of emotions involve important implications for child development (Kårstad et al., 2013).

Emotion-focused therapy (EFT) is a humanistic–integrative approach that emphasizes the significance of human emotions in psychological functioning and therapeutic change (Greenberg and Paivio, 1997). With its early roots in humanistic, Gestalt, and existential therapies (Perls et al., 1951; May, 1977; Yalom, 1980), family system theory (Bowen, 1966), and later research in affective neuroscience (Damasio, 1999; Le Doux, 2002; Schore, 2003), an emphasis on experiential engagement and felt emotions is seen as the primary vehicle of change. From the perspective of EFT, a person must experience painful feelings in order to change them (Greenberg et al., 2008). It is argued that the facilitation of a person’s ability to perceive and emotionally respond to environmental situations in healthy ways yields client improvement in the long term (Pascual-Leone et al., 2016). Process variables that significantly contribute to therapeutic outcomes are empathy, alliance, depth of experiencing, and aroused emotion’s congruence (Pascual-Leone and Greenberg, 2007; Muran and Barber, 2010).

Emotion focused skills training (EFST) is based on research from EFT, person-centered therapy, and humanistic–experiential psychology (Rogers, 1992; Greenberg, 2015). The program originates from emotion-focused family therapy (EFFT; Dolhanty and Greenberg, 2007; Lafrance et al., 2014a) and parental emotion coaching research (Gottman et al., 1996; Havighurst et al., 2013). EFST is a manualized, skills-oriented, and intensive short-term program aimed at parents of children with mental health problems (including preventative parent support); and emphasizes a non-judgmental and resource-oriented attitude toward the parents (Hagen et al., 2019). Parents are seen as both critical and powerful in their child’s recovery (Foroughe, 2018). The program stresses the importance of good working alliance (Bordin, 1994; Watson and Kalongerakos, 2010), congruence, genuine empathy, and unconditional positive regard (Rogers, 1992; Elliott et al., 2011). The program is delivered by trained therapists who explicitly convey their belief in parents’ ability to meet their child’s needs—until they believe it themselves and function as a model–learner in taking responsibility to repair alliance ruptures when needed (Moltu and Binder, 2010; Safran et al., 2011). The program involves training in the four following specific skills: (1) teaching emotion validation, (2) working with parental emotional “traps,” (3) increasing parental capacity to set healthy and flexible boundaries, and (4) repairing relationship between parent and child. The therapists’ ability to take radical responsibility for the therapeutic process and the relationship is considered important for parents’ ability to learn new ways of relating to their child (Dolhanty and Greenberg, 2009). The goal of the program is to increase parental motivation and to develop skills enabling parents to engage in new ways of relating to their child.

Recent studies on EFFT show that the program has an effect on reducing child symptoms, increasing parental self-efficacy (Langeland et al., 2019; Lafrance et al., 2020), increasing parents’ beliefs in their own ability to provide their child with help when dealing with difficult emotions (Dunsmore et al., 2009), reducing parents’ fear to be involved in their child’s recovery, and reducing parental self-blame (Lafrance et al., 2015; Stillar et al., 2016). Nevertheless, only one previous study has conducted an in-depth investigation of participants’ own experiences of receiving such programs (Bøyum and Stige, 2017). In this study, the participants reported increased understanding of their child, increased safety in the parental role, and a better relationship with their child. Since this program’s approach is relatively new, qualitative studies with first-person phenomenological descriptions can add to and further enhance our understanding of what the participants themselves describe as the most valuable parts of the process (Elliott et al., 1999; Levitt et al., 2006). A specific focus on parents’ articulations of their subjective experience illustrated through everyday language may provide a richer description of how they make sense of their change process when receiving this program.

### Aims

The present study aimed to explore how parents experience working with their own and their child’s emotions undergoing a short-term program in EFST that focuses on parental capacity to help their child’s struggle with psychological symptoms and reported changes following their participation in such a program. We explore the following research questions: How do parents describe their perceived changes undergoing the EFST program? What are parents’ views on the effect of the program on their child and on the parent–child interaction?

## Materials and Methods

### Study Setting

Data were collected as part of a larger study, a randomized controlled trial (RCT) in the two largest cities, Oslo and Bergen, in Norway, where the aim was to investigate the effect of this parental program in reducing child symptoms. All parents received the EFST program, which means that both conditions in the RCT contained the basic principles of EFST. Two versions of EFST were compared: one *experiential* containing evocative techniques (i.e., imaginative two-chair dialogues) and one *psychoeducational* condition (with didactic guidance and supervision, without two-chair dialogues). For the RCT, parents were equally distributed and randomly allocated to one of the conditions. Those who were eligible for the program needed to meet the criterion for child difficulties as assessed in a structured intake interview and a questionnaire package. The inclusion criterion was children aged from 6 to 13 years with externalizing and/or internalizing difficulties within clinical range. Children were diagnosed in accordance with the diagnostic criteria of the Brief Problem Monitor—Parent form (BPM-P), which is designed to assess children’s psychopathology symptoms as perceived by parents/caregivers (Achenbach et al., 2011). Families were recruited from public health care services and communal family health clinics and through web advertisements. A total of 313 parents (77 fathers and 234 mothers) of 236 children met the inclusion criteria and completed the entire program.

### Intervention

The program comprised a 2-day group training followed by 6 h of individual supervision. Couples were supervised together. The group format was delivered by two therapists with 20–36 participants, and the supervision was provided every 3–4 weeks. The first individual supervision was provided within 3–4 weeks after the group training and with a maximum of 4 weeks between each supervision. The program and supervision were provided within a 12-week time frame. The major goal of the program was to increase parents’ understanding of their own and their child’s emotions and their sense of responsibility as leading agents of change for their child’s suffering. Specific emotion-focused skills, in addition to facilitation and deepening of emotional experience, were emphasized to increase parents’ sense of competence and belief in their ability to influence their situation.

### Therapists

Eleven clinical psychologists and two family therapists delivered the program. They had 5–15 years (Myears = 10.4, *SD* = 3.8) of clinical experience. All therapists were trained and certified in EFST, involving a minimum of 4-day group training, 15 h of supervision, and at least 1 year of experience using the program. All the therapists received a minimum of 10 h supervision from EFST trainers during the study. There were two male and 11 female therapists.

### Researchers

The first author is a clinical psychologist with 15 years of experience and 5 years of EFT practice and is certified in EFST. The author is specialized in clinical family psychology and is a Ph.D. student. The second author is a clinical psychologist and associate professor with 11 years of experience. He has training in humanistic–existential therapy and mindfulness-based therapy, as well as 8 years of EFT practice. The third author is a clinical psychologist with 13 years of clinical experience and is an associate professor. She has training in humanistic–experiential, relational, and phase-oriented trauma therapy. The fourth author is a clinical psychologist with 25 years of experience and a professor with a special interest in qualitative approaches to studies of personal change processes. He has training in EFT, psychodynamic, and mindfulness-based approaches to therapy. The last author is a clinical psychologist, researcher, author, and trainer in EFT with 15 years of experience.

### Participants

As we were particularly interested in the experiential condition, only parents from this condition were invited to participate in the qualitative part of the study. A text message was sent to all parents who participated in the experiential condition at the same time and shortly after completion of the whole program. The recruitment process was terminated as we reached the sufficient number of participants. A total of 16 parents responded, of whom one parent withdrew afterward. Besides, one interview was lost due to technical problems, thus leaving a total of 14 participants. Six men and eight women aged between 36 and 51 years (*M*age = 39.5, *SD* = 4.4) were interviewed for this study. Eight of the parents in this sample (53.3%) were partners. Five were full-time or part-time students, six had completed 4 years of college/university degree, and three had completed their masters (*M* = 3.7 years). Three were single parents, and 11 were married or cohabitants. The participants were recruited from the two largest cities in (Bergen and Oslo in Norway). Eight participants (57.1%) had received earlier treatment for their child’s mental health issues (1–7 times), and six participants had never received any psychiatric treatment prior to this program.

### Methodological Approach

We aimed to explore parents’ experiences of their own processes while undergoing this parental program by utilizing an open and flexible strategy in the collection and analyses of data. We were particularly attentive to the experiential horizon of the participants since we all have our own presuppositions and beliefs that form our own horizon (or sphere) of understanding (Gadamer, 1975). In order to explore the first-person perspective on relational change, a reflexive thematic analysis approach was employed where we proactively explore our self while initiating a dialogue with the participants and use each participant’s presentation of oneself to help revise our preunderstanding as we make sense of the phenomenon anew (Alvesson and Sköldberg, 2000; Braun et al., 2019). This was embedded within a combined hermeneutic–phenomenological framework since we were both interested in parents’ experiences and the way in which our own perspectives would influence the analyses (Laverty, 2003). The hermeneutic process starts from how the person seeks to understand and gives language to the internal process (Gadamer, 1975). The interpretational process is a fusion of the dialectical interaction between the interviewee and the expectations of the interpreter, which gives meaning to the text and is influenced by the historical horizon of both parts (Polkinghorne, 1984). Phenomenology focuses on the structure of experience and the way in which the person gives meaning to their lived world (Gadamer, 1975). Furthermore, the participants’ descriptions of their lived experience and their pre-understanding are influenced by their social and historical contexts (Heidegger, 1962). The interpretation is both needed and unavoidable when we seek to understand and give meaning to the descriptions provided by the participants (Binder et al., 2012).

### Data Collection Method

The qualitative interviews were based on a semi-structured interview guide that focused on parents’ descriptions of the problems that lead them to seek help, their expectations of the program, their experience of participating in the program (both group training and supervision), and their experienced changes following their participation in the program, including descriptions of their everyday life functioning (how they deal with their own and their child’s emotions and how they perceive the parent–child relationship). All participants signed an informed consent form prior to the interviews. The participants were interviewed between March and April 2019 and shortly after the program completion. The interviews were conducted by the first, second, and third authors who were all clinical psychologists. The interviews lasted from 60 to 90 min, with most lasting about 90 min. The interviews were conducted and transcribed in (Norwegian) language. Quotes in the findings section were first translated by the first author and then examined by the other authors. The interviews were audio-recorded and transcribed verbatim by five graduate students who were supervised by the first author.

### Data Analysis

All the transcribed texts were critically studied by alternating between being close to the text, line-by-line analyses of each participant in a bottom-up process with particular attention paid to the participants’ own wordings (Binder et al., 2012), and having a reflexive theoretical understanding (top–down) for the co-creation of meaning in collaboration with the research team (Finlay, 2002). The reflexive thematic analysis approach was conducted through the following procedure:

(1) All interviewers (the first three authors) shared and discussed their immediate responses and experiences as soon as they completed the interviews with the whole research team.
(2) All authors undertook an initial reading of the transcripts to form an overall picture and to obtain a basic sense of each parent’s experience of receiving the program, thus staying close to the parents’ own descriptions of the useful and difficult aspects of the program (bottom–up). All authors took individual notes about what stood out as the most essential changes in their everyday life and that caught their interest. Meaning patterns, themes, and analytical foci were developed using thematic analysis. We landed the preliminary focus of analysis and formed some tentative themes.
(3) To this end, the first author had a new round of close reading and coded the data described in each interview in accordance with what the research team agreed upon through the use of NVivo 12 (QSR International Ltd., 2017) and shared this analysis with the research team.
(4) All researchers had a new meeting where parents’ responses were critically examined and compared through collaborative dialogue and a reflexive exploration of the contexts of both the researchers and the participants. Possible thematic structures were discussed to enhance and refine the analysis and to formulate the findings, moving back and forth between researchers. Suggestions for thematic structure were shared based on the coded data in an attempt to identify core experiences and meaning patterns until we found a structure of presentation that reflected the parents’ experiences. As a result of this analysis, three main themes were established and formulated through three narratives.
(5) The first author conferred and sorted the data in terms of the three themes and subthemes (top–down), cross-checked whether the material is close to the parents’ own description, and rearranged the units of meaning agreed upon.
(6) A new set of meetings was scheduled where the new sorting was discussed and further nuanced until consensus was achieved.

### Ethics Statement

The study was approved by the Regional Committee for Medical and Health Research Ethics (2018/754/REK sør-øst). The participants signed an informed consent form before starting the program. They were provided with written information about the study, and voluntarily, informed consent was obtained from all of them before joining the program. The data were handled and stored safely in accordance with the regulations from the committee of ethics. All interviews were conducted after program completion. The interviewers were aware of the necessity of creating an environment of safety and trust in the interview setting.

## Results

Data analysis of the participants’ reported experiences resulted in three main themes: (1) “Coming home” as a parent, with two subthemes: (a) New ways of being with their child and (b) Parents’ painful inner world; (2) Reclaiming parenthood—applying new tools and new learning in challenging situations; and (3) This is us—changing the heart of the story. These themes can be divided into three different entrances toward change, where theme (1) illustrates the participants’ general attitudes regarding *being* a parent (“I meet myself with acceptance and I am less afraid”), theme (2) illustrates that they are *doing* things differently as a result of concrete learning and the discovery of something new (“I am meeting my child with acceptance, and something unexpected is happening”), and theme (3) illustrates how they are *living* differently as a result of changed family relationship dynamics (“I experience myself and those around me differently”). We will describe these themes in more detail below. *General results* apply to all participants, *typical results* apply to at least half of the participants, and *variant results* refer to two to three of the participants (Hill et al., 2005).

### “Coming Home” as a Parent

This first theme was a general result where the participants described their experience of being a parent in new ways. They were experiencing their inner lives as being calmer and more relaxed and were more aware of their bodily felt wisdom and cleverness.

#### New Ways of Being There With Their Child

All participants shared examples of how they felt calmer and less reactive as parents:

It is always so much strange going on with my son, he has so much energy! And he starts to jerk around…like he can read, but when I want him to read out loud, he starts to read wrongly on purpose…and I think, ‘No! What in the world is going on with you, right now?’ And then it ends up with us just having fun. The tricky things don’t stick to me that long anymore. It isn’t like he is always naughty, difficult, or challenging. The reading situation doesn’t get better if I shout out: ‘You’re a crappy asshole.’ I manage to change over faster and restore our relationship.

Most of the participants described how they had regained confidence and allowed themselves to be the parent. Getting back into the spotlight, they shared having sufficient competence to guide their children. They described how they returned to something or reentered parenthood: “I can be there!” An attitude of security and self-confidence seemed to be awoken. They stepped into the parental role and described how they managed to be the parent they wished to be: “I thought they needed something else,” as they did not know they could get back in there and take charge. This attitude was related to feelings of confidence and pride:

I have what it takes to be the best parent for my child. I feel wiser, I am his biggest teacher. I have everything that is needed to succeed. And I think every parent need this. This self-confidence. Mm, yeah, it’s a kind of a type of detox, in a way. Or like ‘Ha, this is easy!’

The above quote describes how they had been able to regain inner safety and calmness and to be in contact with their intuition. Many participants described their trust in their child’s ability to deal with and make their own choices and how being calm and showing safety made it easier for them to connect with their child:

My son left school, and his teacher called me to tell me what he had done. I would usually have listened to the teacher and react to my son by saying: ‘What have you done? You need to get back to school right now and apologize!’ or ‘We should change school.’ I am always so fast on the ball! Now, I can sit back and relax. I chose to leave the teachers’ story and listened to my son. And I was able to ask him more openly about how he experience the situation. He started crying and told me what happened. And then, I suggested that this may be linked to the incident that his grandma died recently, and he might feel sad and was missing her. He calmed down and talked to me about it. Like he in a way was trying to sort out the experience by himself. You see, now I have the safety in knowing that he is actually doing well at school and I think that he senses it from me, that I am safe.

Most of the participants described that they discovered practical wisdom as parents. They regained access to their intuitive bodily felt parental voice that they knew they had within them, but somehow lost contact with along their way. Their attitudes regarding their basic understanding were characterized by an increased awareness of their surroundings:

I have become more open to take in other senses and impressions.

I am more conscious about what is happening around me.

They became more attuned, could stay with their children for a longer time, and were more practical and relaxed. Most of the participants described being less preoccupied with their own thoughts, feelings, and shortcomings and were more hopeful about the future. They also described how meeting their own emotions with acceptance helped them to provide the comfort and support that they needed from themselves to be emotionally present for their child:

I am less concerned about how it will go with them in the future or overthinking about: ‘what in the world is wrong with them.’ I have been so scared and shameful to recognize this. I haven’t been able to meet my child’s fear because of my own fear of doing it worse. Now, I try to accept my own feelings, also take them into account, and give myself what I need. I notice that I don’t get that easily affected by my children’s sadness and vulnerability when they are angry or disappointed about something. I feel safer in knowing: ‘Ok, here comes a conflict, but I can stand this!’ I can keep myself calm and know within myself that it will be okay. I am noticing that the situation doesn’t escalate to something unmanageable. I am not that afraid anymore. I really got this! The kids are all right, and it is going to go well.

#### Parents’ Painful Inner World

Working with their own emotions was by no means an easy task. Many parents described their struggles to handle painful insights, challenging encounters, and difficulties experienced on their way. Some parents could not make it to the top to obtain an overview of their situation and were left with feelings of frustration and hopelessness. Variant results implied that it was hard to see their own emotions so clearly, and some participants felt overwhelmed and exhausted after these processes:

After working with my own emotions, I went home and lied down on the coach for 1 h. Ah, I think I might be in contact with […], like my body is telling me that I needed to relax. So, I did some restoration before I picked up my child from school. I felt like I was left alone with open wounds.

However, most of the parents who described working with their emotions as really intense and painful were left with feelings of relief. The participants described acquiring insight about how their own emotions sometimes came in the way of meeting their child. The process was heavy but relevant and helpful:

It felt like being on a rollercoaster. I remember working with the feelings I don’t like in my kids. It was so hard to admit out loud what I don’t like. And then I realized: ‘but this is my problem!’ I remember feeling smaller and smaller and smaller. You just want to disappear […]. It becomes so clear how it must be for your child […], what can I say […] to receive this dismissing attitude from you. It was really difficult. I remember feeling completely exposed. It felt like lying on the operation table, naked, and totally without any defense. To see yourself so vulnerable, so clearly […]. We have broken the shell of some things, and it is soft inside but still open. It hurts but also feels good in a way. It’s like […] aah, a relief […] to finally get there.

During this process, some of the parents described how they reached out to their own inner child and how it was for them to grow up in their own childhood. They also mentioned that they discovered sides of their own parents within them:

I remember when I discovered my own mom within me […], the same feeling came up in me that I used to feel when I was a little girl. That experience […] was by far the most painful for me. Not only was I reacting to my daughter like my mom used to, but feeling so vulnerable, experiencing the same hurt in the belly, and being frustrated to never be understood […] was really intense. I grew up with a mom that never recognized my feelings. And those things have kind of turned back inside me. That I never was met in the way I am trying to meet my child. I never learned this. That my daughter might experience me the same way I experienced my mom. And then, there is this blaming of myself, like: ‘why didn’t I know this?’ This is something every parent does!

Some parents described being surprised about getting something unforeseen out of it:

It comes to you as a light in relation to how it is for your child to, what can I say […], take in your being in a way. How they perceive you […], and that is really not funny. It is horrendous. Had I not been challenged; I might never have seen this! I always thought I was sympathetic! This wasn’t the reason I signed up for this program. I wanted to fix my kids.

### Reclaiming Parenthood—Applying New Tools and Learning in Challenging Situations

The second theme was also part of the typical results. Parents described doing what was needed to master their parental role, taking control back and responsibility for the situation by not giving up their role as parents. A sense of agency had found place, and parents’ subjectively lived experience showed how they acted differently and took active initiative toward their children. By employing the learned skills, they noticed that something unexpected happened. Therefore, they personalized the tools to better fit into their everyday life and used it creatively. One mother described how acknowledging her daughter helped her to move forward:

I went to the grocery shop with my daughter. I was in a hurry, and my daughter wanted everything inside the store. She pointed at some frozen shrimps, it was in the middle of the winter, and said she wanted them. I continued saying no, no, no. Suddenly, I remembered what I had just learned. So, I just jumped into it. I am not sure what I expected […] but decided to give it a try. So, I sat down and said: ‘I understand you want the shrimps. Ever since you were a baby you have loved shrimps. Do you remember the picture where you are sucking on the shrimps? You want them because they are salty and tasty and remind you of the summer, right?’ And then it just clicked. Before I knew it, she turned her face around and pointed at some brown cheese: ‘Look mommy, this is the same we have at home’ and forgot everything about the shrimps! Suddenly, we were back on track and continued shopping. I was amazed and thought: ‘Did you guys pay her?’

This mother seemed surprised and did not expect to obtain such a reaction from her daughter. Many parents described that they were trying to meet their child’s feelings with acceptance without having the urge to cheer them up, use diversion, or help them to see the brighter side:

My son has reading difficulties, and I yelled at him. Then I tried to imagine how it might have felt for him, so I said: ‘I can see that this is difficult for you and that you are upset. No matter how hard you try, it is still difficult for you to read. And then I started messing with you. That wasn’t fair. No wonder you got angry at me! And I think you might have had it like this for quite some time.’ He broke down completely and busted into tears. It turned out that he was having these difficulties since he started school. And this loosened up pretty much for us.

Not every parent found this way of acknowledging their child’s emotions as helpful: “[…] I think he appreciated that we spent more time in the evenings, talking more about his feelings than we used to. But he didn’t get any better.”

Typical results show how some participants tried to apologize for things that have happened in the past, even if they could not change the situation. By doing so, they discovered that a lot of feelings rose in their child, and they were able to talk about it:

I apologized to my son for the divorce. I remember that he was angry and upset at the beginning, but over time he became emotionally flat. He disappeared into the gaming world and pretended like everything was totally fine. And when I apologized that I and his dad were not able to live together as a family and how hard it must be for him to live biweekly at dads and my place, I expected him to be that nice son he always is by telling me: ‘It’s fine mom, I understand why it had to be this way.’ But I held on the apology. And then he started crying and shared how painful this was for him. He told me: ‘This is so difficult for me […].’ A lot of feelings came up in him! He usually doesn’t cry […] but it felt like something clicked into right place.

Yet, many parents shared how difficult, energy-demanding, and time consuming it could be to choose the right thing. Even if the techniques seemed effective and useful, the parents sometimes lacked the courage or motivation to do the intense work and came up in situations where they got really angry at their kids: “It is not ok to spit on the floor!” However, the majority of the parents described that they could still feel an inner safety even if they were not able to use the skills in the moment, just knowing that they could always go back and repair:

I have a technique at hand now. It is a safety just knowing it. And if you haven’t been able to follow it in the heat of battle, you can always go back and repair […]. The relationship is not ruined even if there has been a lot of anger and frustration. It can still go well in the future. […] I have regained belief that I can actually help my kids, that I have something to contribute with.

Knowing they had the skills required to react differently gave them a lot of responsibility, but also hope, believing they were capable of providing their children with the help they needed.

### This Is Us—Changing the Heart of the Story

The third theme was also part of the general results. Participants shared that their family dynamics were somehow changed. They described perceiving, experiencing, and talking about their family differently. Their narratives and their relational understanding of who they are as a family were altered, and they described becoming aware of the sides of their loved ones they had previously ignored. Changing the story about who they are altered the story about their partner, child, and family.

Participants described how they were forming a new story about their children. They reported developing a more nuanced picture of their child’s inner workings and described giving them more space and trusting them more. One mother described how she was trying to understand her son as an independent human being and was reaching toward him with an intention to understand:

I can look at him more positively and enjoy more of his person. This is what it is all about; we need to look more at him and think, ok, this is who he is! And instead of criticizing him for being too excited or too much, we are actually having more fun together.

They described being able to perceive their child differently, trying to appreciate more of who they are:

I don’t mess that much with him that he should be able to get more friends. I now get that he doesn’t need more friends. He has one good friend and some other friends. And for him, that is more than enough! I can see that he is not alone, right? I understand more of who he is, and I think he feels that I have a better understanding of […] what’s really going on.

A few parents described their children as being more responsible by sorting out difficult situations on their own. The parents realized that they did not have to fix all their problems:

Our daughter was experiencing some difficulties at school. And when I opened up and listened more to what she might experience as difficult, she told me that she felt ignored at school. It felt good that she could come to me with this. This is something I also want! I treat her more as a mature. And she can come to me when she feels that she needs to, without me commanding things. The difficulties are still there, but I can see there are growing parts of her that is peaceful and are doing well. This is also her. And we can talk more peacefully about situations that occur.

Variant results showed that participants described appreciating more of who they are as a family than being concerned about how other people might perceive them: “Like my kids can sometimes get dessert before dinner or sometimes drop the night brush. The children feel like: “Gosh, what a cool night this became!” Many participants described how meeting the child in concern with acknowledgment helped them to show more empathy toward their siblings. One participant described:

We went to a restaurant with my daughter, and she got this large mango lassi. She wasn’t able to drink up, so we brought it back home. My son loves mango lassi, so when he came home after training, he opened up the refrigerator and said that this is his! I said: ‘This is your sister’s and she really wanted it.’ I saw his angry face as he was reaching toward the glass […]. And you know, as part of this process, I have realized he might have experienced his younger sister as a threat in his life. So, I just started to respond: ‘I understand that you might have also wanted this, this is something you really love. And now she got this and not you! No wonder you feel that this is unfair! And you might even feel that I love her more than I love you because she got this and not you.’ I saw that his face turned softer and more vulnerable, so I responded: ‘You know what, we can share it, so you also get the chance to taste it!’ After doing this for a while, he responded: ‘No, it’s ok. This is hers. She can have it,’ and put it back in the refrigerator. I didn’t know he could be this nice big brother!

This story illustrates not only “reclaiming parenthood” since this father is actively using the new skills but also his changed conceptualization of his son as he realizes that he “might have experienced his sister as a threat in his life.” Furthermore, it illustrates how being met with generosity helps his son to let go of his needs and to show generosity toward his younger sister. Most of the parents also described more empathy toward their partner and felt being together in it:

It felt really strong to witness my partner’s pain when she told what she was struggling with inside her. I got really emotional when she said that she is trying to be there for everyone else, but no one was there for her. We have really been struggling in our relationship […] and we have spent less time to get deeper in the core of our pain. We have witnessed each other’s pain, and it has been as effective as working with my own. We have started to talk more about this at home, and I feel that we have become closer. I can now share my true feelings with her, and we can talk about what is really going on. It is easier for me to be there for her. And she can understand and be there for me. We are standing in it together.

Witnessing each other’s emotional pain had been as effective as working on one’s own. This process seemed to have helped them to find opportunities that they did not know were there. They described facing the situation with their children together as a family. They were redefining relationships and were seeing themselves and their children through a new lens.

## Discussion

In this study, we explored parents’ experiences working with their own and their child’s emotions following their participation in a skills-oriented and emotion-focused intervention, the perceived changes in their everyday life, and their understanding of their child’s difficulties. Our findings suggest three core themes or change entrances that capture the participants’ experiences. The first theme, “Coming home” as a parent, showed how the participants gained access to their practical wisdom as parents. Something happened inside of them, and their inner life had changed. They described having an inner wisdom, bodily awareness, calmness, and being less afraid; they were meeting their own and their child’s emotions with acceptance. Many parents described that they did not know they had it in them, and this changed attitude of willingness to do what is needed provided them with an inner security and self-confidence, like coming back home again with a more focused, capable attitude, and a desire to do the right thing. They further described working with their own emotions as being both demanding and helpful. In the second theme, “Reclaiming parenthood,” parents described trying out the skills they learned, experienced that it worked, and described emerging feelings of self-efficacy and agency. However, they also reported to have noticed that something unexpected happened. This element of surprise motivated them to employ the techniques creatively based on what was needed and to personalize the tools to better fit them into their everyday life. In the third theme, “This is us,” parents described how they had gained a new relational understanding of their children, partner, and family that they had previously ignored.

The findings from the first two themes were in line with the general trends in previous research in EFT (Greenberg and Pascual-Leone, 2006). Both themes seemed to influence each other; meeting oneself with acceptance and being less afraid can help to meet your child with acceptance, and trying out the skills on your children and noticing something unexpected can help you gain confidence that you have what it takes to use the learned skills creatively. Moreover, working with their own emotions was experienced as intense and painful. Nonetheless, it was helpful and considered as a relief. These findings are also in line with those of previous qualitative research on EFT, such as Lafrance et al. (2014b), Stiegler et al. (2018), and Nødtvedt et al. (2019), indicating how the establishment of trust is an important precondition when working with painful emotions and that participation in such interventions is related to increased awareness of agency. Moreover, the presented findings are in line with previous qualitative research on EFFT, indicating the link between increased understanding and the acceptance of parents’ own and their child’s emotions (Bøyum and Stige, 2017; Foroughe, 2018). Previous studies have indicated that successful psychotherapy hastens change by enhancing the contribution of hope and agency as a common factor (Miller et al., 1999; Snyder, 1999). People are likely to experience positive emotional responses and maintain hope when they are able to both pursue their goals and generate alternative pathways when needed (Elliott et al., 1991). These findings can also be understood as changes in schematic processing of emotion, as if the parents have been helped to change and apply adaptive and congruent emotion schemes. The principal target of change in EFT is on accessing primary adaptive feelings through working with maladaptive emotion schemes in order to make them amenable to change (Greenberg and Pascual-Leone, 2001). With a wider look, the findings can also be seen from the perspective of social learning theory, as in Bandura’s (1971) concept of self-efficacy that addresses the importance of parental beliefs about their own efficacy and mastery. However, in order to understand the emerging development of motivation, desire/need, or willingness in addition to practical and flexible use of learned skills, the term “practical wisdom,” as described by Schwartz (2011), seems to be a better fit. According to Schwartz, practical wisdom is having the moral will to do the right thing and the moral skill to figure out what the right thing is in any particular situation. Although rules and incentives, such as in social and behavioral learning theory, may be well intended, they cannot do the job in any situation that involves human interactions in the way that character traits, such as practical wisdom, can (Schwartz, 2011).

In addition, parents perceived pain while undergoing this program, and their descriptions of fear and shame could indicate that the program had emotional relevance. In emotion-oriented theories, such as the affect integration theory and EFT, the moral will stem from inner emotions or affects, which motivate us (Nathanson, 1992). Our findings suggest that parents seem to become emotionally convinced about their important role as parents. Their motivation seems to be derived from a growing emotional desire or need to be there for their child. Here, affects can be understood as the engine that motivates them: “The need created by a drive, can be assembled with an affect to make the function more urgent and give it motivation (…). Whenever we are said to be motivated toward what is important to us, it is because the affect has made us so” (Nathanson, 1992, p. 89). Parents’ descriptions of self-confidence and pride can also be related to their needs created by the affects: “pride itself is infectious, both to the one who has suddenly experienced efficacy and to those watching” (Nathanson, 1992, p. 84). Hence, pride, agency, and self-confidence can be understood as the antidote of fear and shame. Besides, parents’ descriptions of becoming aware of and listening to bodily and emotional signals, arising from both themselves and their partner and child, and finding ways of caring for themselves are also considered as important in mindfulness-based treatments (Kabat-Zinn, 2003; Hjeltnes et al., 2016). The process of meaning construction is an ongoing dialectic synthesis between conceptual symbolization and reflection as well as emotional arousal and bodily felt sense (Gendlin, 1996). Bodily awareness helps in discovering agency to change and being able to do something to influence their situation—as an antidote to loss of control (Stige and Binder, 2016).

However, many participants described working with their own emotions as quite painful, and a few described the emotional intensity as overwhelming. This was also found in Stiegler et al. (2018) and does not come as a surprise, considering the intensive quality of this therapeutic approach. This theme shed light on the tremendous effort put in by the parents when participating in such programs. Additionally, these findings can be explained by the human tendency to avoid painful feelings (Foa and Kozak, 1986). Some participants described their lack of motivation to choose the right thing. This can be understood as the phenomenon “competing motivation” principle used in motivational interviewing (Miller and Rollnick, 2012). In maladaptive emotions, we react to the past in the present (Greenberg, 2015); even parents who are strongly motivated to help their kids can get caught in old fair, shame, and avoidance. Variant results showed that some participants were confused, which can be explained by the short-term quality of the program. Some participants might have needed more time to consolidate the process.

Some of the findings from the third theme, “This is us,” are in line with EFT for couples (Goldman and Greenberg, 2007) and EFT (Girz et al., 2012; Strahan et al., 2017). Parents were simply generalizing their learning from one child to other family members, including their relationship with siblings. Interestingly, we did not expect that the participants would report changed relationship dynamics and narrative understanding of the other children (not only the child in concern) and between siblings. These findings concur with the theoretical perspective of structural narrative therapy, which is based on the assumption that people experience problems when the stories of their lives do not sufficiently represent their lived experience (White and Epston, 1990). In this tradition, therapy becomes a process of re-storing a person’s life and experiences, encouraging a sense of authorship of one’s experiences and relationships in the telling and retelling of one’s story (Anderson and Goolishian, 1992). In such structural family therapy traditions, the goal is to conceptually restructure the family organization, moving away from an understanding of the child as the one to blame to the whole family system as being dysfunctional (Minuchin, 2012), and to mobilize the relationship’s strengths in order to make disturbing symptoms unnecessary or excessive (Barbetta, 2017). Nevertheless, this may not reflect the case for all parents. From an EFT perspective, participants’ altered family narratives and changed relationship dynamics can be understood as the process of client storytelling through an emotional differentiation and meaning making process called dialectical constructivism (Angus et al., 2012). In this perspective, parents’ changed family narratives can be understood as an ongoing dialectical reconstruction of meaning through conceptual and emotional processing. Parents made sense of their experiences through a reflexive awareness and meaning making process, alternating between emotion experiencing and making sense of these experiences.

## Reflexivity

The perspective or position of the researcher shapes all the research, quantitative or qualitative. Three members of the research team are trained in EFT, all five researchers are quite familiar with the method, and three of the authors are trained in EFST. Researcher allegiance is as such an issue to be concerned with. Even if some degree of researcher bias or skewedness in this study was unavoidable, awareness about preconceptions and openness toward the examination of how the subjectivity and perspectives of the researchers may inform different stages of the research process can compensate for this imbalance (Finlay, 2002). In this study, we considered it important to be aware of how the professional background of several members of the research team might inform the research process. This may pose a limit to a more neutral position, but might even lead to a richer and different yet equally valid scope of perspectives and more developed understandings of phenomena (Barry et al., 1999). The first author conducted five of the interviews and played an essential role in developing the transcript and handling the data. To address these challenges, the research team included two members that were not affiliated to the EFST approach and made an explicit effort to be reflexive about how the analyses may be influenced by our preconceptions.

## Limitations

There are some limitations in the transferability of the results from this study that are important to address. The participants were interviewed right after the program completion, which might have contributed to simplifying accurate recall of events. Nevertheless, participation in the program was voluntary, and most of the participants were from an ethnic (Norwegian) middle-class population rather than from particularly vulnerable groups such as the child welfare system. In addition, the parents who joined the program were highly motivated prior to the treatment for getting help due to child mental health difficulties. The study investigated experiences from a relatively small sample size of 14 participants (of 313 parents). The selected sample is likely to have contributed to additional bias since parents who most enjoyed and/or benefited from the program are likely to have been most motivated to participate in the interviews. There may be a number of potential differences between this subsample and the larger group of parents who received the program. As this program is normally offered in a private health care clinic and costs money, there is a risk of attributing positive change to the program actually caused by other factors, which could potentially contribute to difficulties in being fully honest about negative experiences during the program in the interview context. Finally, following an interview manual and a specific focus on parents’ everyday life experiences could have led to the interviewers being less flexible in choosing how to respond to participants’ immediate responses. This may represent a limitation in terms of ecological validity.

## Implications for Research and Clinical Practice

The present article has examined parents’ subjective experiences of receiving the EFST program. Further in-depth qualitative studies of participants’ experiences in different contexts are needed in order to examine the transferability of the findings to other contexts. Qualitative studies of immediate parental experiences after receiving the EFST program, by using interpretative process recall or hermeneutic single-case study designs (Elliott et al., 1999), may thus be important to complement this study and describe the complex moment-by-moment processes that contribute to the changes in the child, parent, partner, and relationship dynamics. Further research is needed to investigate how the participants’ family dynamics have changed after receiving the program. The findings in this study indicate the need for therapists to be sensitive and aware of parents’ needs and preferences when working with their own emotions in this way and to normalize expected post-reactions after program completion. Finally, the findings highlight the relevance of the therapist being open to negative feedback and that this open and non-defensive attitude is expressed clearly and unequivocally to the client.

## Data Availability Statement

The datasets generated for this study are not readily available because the dataset is consisting of interview data, confidentiality can not be safeguarded. The data will therefore not be made available. Requests to access the datasets should be directed to NA, ansar@ipr.no.

## Ethics Statement

The studies involving human participants were reviewed and approved by Regional Committee for Medical and Health Research Ethics 2018/754/REK sør-øst A. The patients/participants provided their written informed consent to participate in this study.

## Author Contributions

NA wrote the first draft of the manuscript. NA, JS, AH, and SS contributed to the conception and design of the study. NA led the analysis of the data. NA, JS, AH, SS, and P-EB contributed to the analysis of the data and to sections of the manuscript. All the authors contributed to the manuscript revision and read and approved the submitted version.

## Conflict of Interest

NA and JS discloses a conflict of interest because they work in an organization that conduct training in the method. The remaining authors declare that the research was conducted in the absence of any commercial or financial relationships that could be construed as a potential conflict of interest.
